# Aqueous Extract of *Psiloxylon mauritianum*, Rich in Gallic Acid, Prevents Obesity and Associated Deleterious Effects in Zebrafish

**DOI:** 10.3390/antiox11071309

**Published:** 2022-06-30

**Authors:** Batoul Ghaddar, Laura Gence, Bryan Veeren, Matthieu Bringart, Jean-Loup Bascands, Olivier Meilhac, Nicolas Diotel

**Affiliations:** 1 Université de La Réunion, INSERM, UMR 1188, Diabète Athérothrombose Thérapies Réunion Océan Indien (DéTROI), 97400 Saint-Denis, La Réunion, France; batoul.ghaddar@univ-reunion.fr (B.G.); laura.gence@univ-reunion.fr (L.G.); bryan.veeren@univ-reunion.fr (B.V.); matthieu.bringart@inserm.fr (M.B.); jean-loup.bascands@inserm.fr (J.-L.B.); olivier.meilhac@univ-reunion.fr (O.M.); 2 CHU de La Réunion, 97400 Saint-Denis, La Réunion, France

**Keywords:** blood-brain barrier, neurogenesis, obesity, oxidative stress, *P. mauritianum*

## Abstract

Obesity has reached epidemic proportions, and its prevalence tripled worldwide between 1975 and 2016, especially in Reunion Island, a French overseas region. *Psiloxylon mauritianum*, an endemic medicinal plant from Reunion Island registered in the French pharmacopeia, has recently gained interest in combating metabolic disorders because of its traditional lipid-lowering and “anti-diabetic” use. However, scientific data are lacking regarding its toxicity and its real benefits on metabolic diseases. In this study, we aim to determine the toxicity of an aqueous extract of *P. mauritianum* on zebrafish eleutheroembryos following the OECD toxicity assay (Organization for Economic Cooperation and Development, guidelines 36). After defining a non-toxic dose, we determined by liquid chromatography coupled to tandem mass spectrometry (LC-MS/MS) that this extract is rich in gallic acid but contains also caffeoylquinic acid, kaempferol and quercetin, as well as their respective derivatives. We also showed that the non-toxic dose exhibits lipid-lowering effects in a high-fat-diet zebrafish larvae model. In a next step, we demonstrated its preventive effects on body weight gain, hyperglycemia and liver steatosis in a diet-induced obesity model (DIO) performed in adults. It also limited the deleterious effects of overfeeding on the central nervous system (i.e., cerebral oxidative stress, blood-brain barrier breakdown, neuro-inflammation and blunted neurogenesis). Interestingly, adult DIO fish treated with *P. mauritianum* display normal feeding behavior but higher feces production. This indicates that the “anti-weight-gain” effect is probably due to the action of *P. mauritianum* on the intestinal lipid absorption and/or on the microbiota, leading to the increase in feces production. Therefore, in our experimental conditions, the aqueous extract of *P. mauritianum* exhibited “anti-weight-gain” properties, which prevented the development of obesity and its deleterious effects at the peripheral and central levels. These effects should be further investigated in preclinical models of obese/diabetic mice, as well as the impact of *P. mauritianum* on the gut microbiota.

## 1. Introduction

Obesity is a global epidemic, characterized by an excessive accumulation of body fat. The World Health Organization (WHO) has estimated that over 1.9 billion adults are overweight, including 650 million obese people [1]. Obesity leads to many physiological complications, impairing body homeostasis through increased chronic inflammation and oxidative stress [2,3,4]. Thus, obesity can affect the cardiovascular and renal functions, and promotes liver steatosis and insulin resistance, among other complications [5,6,7,8]. Thus far, obesity represents a major risk factor for the development of type 2 diabetes and its complications [9].

In addition to these peripheral disorders, it was also shown that obesity alters brain homeostasis and leads to a variety of central nervous system (CNS) alterations [10,11]. For instance, obesity promotes stroke, blood-brain barrier breakdown, neuro-inflammation, as well as oxidative stress. It also results in cognitive impairments (memory defects and dementia) and in increased risks of developing neurodegenerative diseases [12,13,14,15,16]. In addition, it was recently demonstrated that obesity worsens brain plasticity by decreasing neural stem cell proliferation and impairing other neurogenic processes [17]. 

Nowadays, the scientific effort is focusing on establishing successful pharmacological treatments to limit weight gain and/or to limit the deleterious effects of obesity on the central nervous system [18,19]. Medicinal plants are envisioned as a natural storehouse of beneficial compounds including alkaloids, flavonoids, tannins, essential oils and phenolic compounds [20], which could help to combat obesity or its complications. For this reason, it is important to screen the potential beneficial effects of medicinal plants to prevent the onset of obesity or obesity-induced complications. Reunion Island is a biodiversity hotspot known for its wide distribution of endemic plants. Some of them are traditionally described and used for their suggested “anti-diabetic” and/or lipid-lowering properties [21,22,23,24,25]. Unfortunately, scientific data are lacking to confirm such properties, although recent data from our group document some of these beneficial effects [25,26,27,28,29,30,31]. Recently, 27 Reunionese plants have been registered in the French pharmacopeia [21,22]. Among these medicinal plants, *Psiloxylon mauritianum* (*P. mauritianum*; vernacular name: bois de pêche marron) is traditionally used for the treatment of diarrhea and to decrease the formation of uric acid in the body (gout pathology) [32]. Other studies have also reported the antiseptic, antispasmodic and diuretic effects of *P. mauritianum* [32]. Of interest, *P. mauritianum* is reported to exhibit “anti-cholesterol” and “anti-diabetic” effects, highlighting its potential beneficial role in metabolic diseases [32,33]. In the literature, the presence of flavones, flavans, flavonoids, phenols, terpenes and tannins was shown in the acetonic extract of *P. mauritianum* leaves [34]. Checkouri and colleagues have further characterized the polyphenolic composition and antioxidant capacity of *P. mauritianum* [25]. 

Recently, zebrafish has emerged as an alternative model to study metabolic disorders, including diabetes and hyperglycemia, as well as overweight/obesity [29,35,36,37,38,39,40,41]. Considering obesity, zebrafish shares several aspects of the mammalian pathology, with high adiposity, increased body mass index (BMI), non-alcoholic fatty liver, the development of insulin resistance and hyperglycemia, cardiovascular diseases, as well as brain complications [29,37,42,43,44,45,46,47]. In addition, zebrafish is also a widely used model to investigate brain plasticity [48,49,50,51]. 

Interestingly, recent data also documented the deleterious impact of metabolic disorders (herein hyperglycemia and obesity) in the brain of adult zebrafish [29,52,53,54,55,56]. These metabolic disruptions induce blunted constitutive and regenerative neurogenesis, as well as cognitive impairments, in a manner similar to mammals [29,52,53,54,55]. Consequently, zebrafish has emerged as an interesting and valuable model to study the impact of metabolic disorders on the central nervous system (CNS). 

In this study, based on *P. mauritianum*’s composition and richness in antioxidants and considering the involvement of oxidative stress in obesity, we hypothesized that this medicinal plant could limit weight gain and the development of obesity complications. We first aimed to investigate the in vivo toxicity of the aqueous extract of *P. mauritianum* in zebrafish eleutheroembryos using the Organization for Economic Co-Operation and Development (OECD) toxicity assay (guidelines 236: Fish Embryo Acute Toxicity). Next, using a model of high-fat-diet (HFD) larvae, we studied the lipid-lowering effects of the aqueous extract of *P. mauritianum* at a non-toxic concentration. Finally, taking advantage of our recently developed obese zebrafish model (diet-induced obesity: DIO) [29], we investigated the effects of *P. mauritianum* on metabolic parameters (i.e., body weight, BMI, glycemia) and brain homeostasis. In this work, we demonstrate the interesting preventive properties of *P. mauritianum* on the development of obesity and its associated complications.

## 2. Materials and Methods

### 2.1. Animals and Ethics

Three- to four-month-old adult wildtype male and female zebrafish (*Danio rerio*) were obtained from our zebrafish facility and were maintained under standard conditions of temperature (28.5 °C), photoperiod (14 h dark/10 h light), pH (7.4) and conductivity (400 μS). Zebrafish embryos were obtained from the crossing of adult animals and were maintained in embryonic medium (E3 as prepared by [57]) at 26.5 °C. All experiments were conducted in accordance with the French and European Community Guidelines for the Use of Animals in Research (86/609/EEC and 2010/63/EU) and approved by the local Ethics Committee for animal studies of CYROI and the French Government (APAFIS_ 20191105105351_v10 and APAFIS_ 2021080209405969_v8). 

Note that the term eleutheroembryos refers to a developmental stage from 0 to 120 h post-fertilization (hpf), when the animal was using the yolk sac, while the term “larva” is used when the animal begins to feed autonomously, approximately after 5 days post-fertilization.

### 2.2. Eleutheroembryo Toxicity Assay

The toxicity tests were performed according to the OECD test guideline 236 [58]. Newly fertilized zebrafish eggs (<3 h post-fertilization (hpf)) were incubated until 96 hpf with different aqueous extract concentrations of *P. mauritianum* (1.87, 1.25, 0.83, 0.55, 0.37, 0.24 and 0.16 g/L), or kept in E3 embryo medium as a control condition. The treatment was carried out on 20 embryos/concentration and renewed every day. Four apical endpoints were recorded daily as indicators of acute lethality: coagulation of the embryo, lack of somite formation, non-detachment of the tail bud from the yolk sac and lack of heartbeat. The concentration of the aqueous extract leading to 50% of death (LC_50_) was calculated and the maximum non-toxic concentration (MNTC) was determined as 0.25 g/L

### 2.3. Larvae Overfeeding Protocol

In order to analyze the potential lipid-lowering effects of the plant extract, we selected 7 days post-fertilization (dpf) larvae, which were divided into 3 groups (CTRL, HFD, HFD + *P. mauritianum;* 20 per group), according to a previously established protocol [31]. The CTRL group was fed with normal food (GEMMA 75), the HFD group with 0.1% egg yolk diluted in E3 medium and the HFD + *P. mauritianum* group with 0.1% egg yolk dissolved in the aqueous extract (0.25 g/L), prepared in E3 medium. The treatment was renewed every day with freshly prepared solution and diet. At 11 dpf, the larvae were anesthetized and fixed for ORO labelling.

### 2.4. Diet-Induced Overweight/Obesity (DIO) Protocol

Adult zebrafish (3 to 4 months) were divided into 3 groups (CTRL, DIO, DIO + *P. mauritianum* at 0.25 g/L). Fish were distributed to each tank at the same density per group (10 to 20 fish maximum per 3.5 L tank, according to the experiment). Adult zebrafish were fed as described previously in [29]. Briefly, the control group was fed once a day with dry commercial food in the morning (15 mg/fish/day, GEMMA 300, Planktovie) and freshly hatched artemia (6 mg/fish/day, Artemia cysts; REF: B052-P) in the afternoon. The DIO and DIO + *P. mauritianum* groups were fed six times a day (at 8 a.m., 10 a.m., 12 a.m., 2 p.m., 2 a.m., 6 p.m.) with dry food (52.5 mg/fish/day) and three times (at 1 p.m., 3 p.m., 5 p.m.) with freshly hatched artemia (30 mg/fish/day) in the afternoon. These treatments were performed in a two-week period in the therapeutic plant treatment and in a four-week period in the preventive plant treatment according to experiments (see below).

### 2.5. Plant Material and Preparation of the Aqueous Plant Extract for Toxicity Tests and Preventive/Therapeutic Treatments

Leaves of *P. mauritianum* (Industrial lot: FLBPM 20180427; GPS coordinate: −20.947334, 55.548979″) were obtained from the Cooperative des Huilles Essentielles de Bourbon (CAHEB). Leaves were dried, crushed and stored at − 20 °C. Aqueous extracts of *P. mauritianum* were prepared every day to renew the treatments performed on eleutheroembryos, larvae and adult zebrafish, respectively.

Briefly, for the toxicity assay in eleutheroembryos, infusion of 0.75 g of *P. mauritianum* was performed with 50 mL of boiled E3 for 10 min under agitation. The solution was next filtered and a serial dilution was performed to obtain the following concentrations: 1.87, 1.25, 0.83, 0.55, 0.37, 0.24 and 0.16 g/L. A similar protocol was used for the treatment of HFD larvae at 0.25 g/L.

To prepare the plant infusion for adults, 0.5 g of crushed plant was infused with 250 mL of boiled fish water for 10 min under agitation. After filtering, 250 mL of *P. mauritianum* extract was added to 1.750 L of fish water to reach a final volume of 2 L (final concentration of *P. mauritianum*: 0.25 g/L). Adult fish were treated with *P. mauritianum* from 6 p.m. to 8 a.m. (5 days a week) during the whole experimental DIO protocol. The overfeeding protocol took place over 4 weeks for the preventive treatment. For the therapeutic treatment, fish were overfed under a DIO protocol for 1 week before being treated with *P. mauritianum* for 1 week. During these overnight *P. mauritianum* exposures, all the groups (CTRL, DIO, DIO + *P. mauritianum*) were maintained outside of the fish system with the same volume of water.

### 2.6. Body Weight, Body Mass Index (BMI) and Fasting Blood Glucose Measurements 

Every week, fish from each group were weighed. After being retrieved using a net and dried on tissue paper, they were briefly weighed and immediately placed back into the water. This procedure lasted around 20 s. In addition, the body length of the fish was measured at the beginning (first day, during the weighing process) and at the end of the experiments in order to calculate the BMI, as previously described [29,54]. Fasting blood glucose was measured using a glucometer, as previously described [41,55]

### 2.7. Tissue Preparation

At the end of the experiments, fish were euthanatized before being fixed in 4% PFA (paraformaldehyde) dissolved in 1X phosphate-buffered saline (PBS). For cryostat sections, the liver was dissected and cryopreserved by overnight incubation in 1X PBS, containing 30% sucrose. Then, they were embedded in an OCT matrix and cut using a cryostat at 12 µm thickness. For vibratome sections, the brain was dissected from fixed fish in 4% PFA for overnight. Brains were kept in 100% methanol at −20°C for immunohistochemistry. For qPCR or protein analyses, the tissues of interest were immediately dissected, snap-frozen and kept at −80 °C.

### 2.8. Oil Red O (ORO) Staining 

For ORO staining in HFD larvae, after euthanasia and overnight fixation in 4% PFA at room temperature, samples were washed and dehydrated. ORO (Sigma-Aldrich; Schnelldorf, Germany; REF: 00625) was dissolved in 60% isopropanol diluted in distilled water (final concentration: 0.15% ORO in 60% isopropanol). The larvae were kept in ORO solution overnight at room temperature. After rehydration and washing in 1X PBS, larvae were finally imaged with a binocular microscope. The staining intensity was measured using ImageJ.

For ORO staining of the livers of CTRL, DIO and DIO + *P. mauritianum* fish, frozen liver sections were rehydrated and rinsed with 60% isopropanol. The liver sections were stained for 40 min with freshly prepared ORO (Sigma-Aldrich; Schnelldorf, Germany; REF: 00625) according to a standard protocol. The sections were incubated for 30 s with Mayer’s hematoxylin for nuclei counterstaining. Finally, the sections were dehydrated and mounted with mounting medium (IMM Ibidi; REF: 50001). After imaging with the Nanozoomer S60 (Hamamatsu; Life Sciences, Tokyo, Japan), the red intensity was evaluated using ImageJ quantification of the red color intensity (plugins > filters > color transformer > Luv > u channel > measure same area).

### 2.9. RNA Extraction and Reverse Transcription

Zebrafish brains were removed from the skull, pooled (n = 2) and stored at −80 °C for later RNA extraction. Similarly, euthanized larvae were pooled (n = 40) and stored at −80°C. Three pools of brain or larvae were ground with a TissueLyser II (Qiagen, Chatsworth, CA, USA) and RNA extraction was performed using the RNAeasy Mini Kit (Qiagen; Courtaboeuf; France), according to the manufacturer’s protocol. Then, reverse transcription for 2 µg of RNA into cDNA using random hexamer primers was performed (ThermoFisher Scientific, Dardilly, France; REF: 100026484), as well as MMLV reverse transcriptase (Invitrogen, France; REF: 28025-021). 

### 2.10. Gene Expression Analysis by qPCR

Semi-quantitative PCR experiments were performed using the Biorad CFX Connect Real-Time System using the SYBR green master-mix (Eurogentec; Rue Bois Saint-Jean Belgium) and specific zebrafish primers. Each PCR cycle was conducted for 15 s at 95 °C and 1 min at 60 °C. Melting curve analyses and PCR efficiency were performed to confirm correct amplification. Results were analyzed and the relative expression of the pro-inflammatory marker Nuclear Factor Kappa-B (*nfκb*) and tumor necrosis factor alpha (*tnfα*), liver toxicity genes fatty acid binding protein 10a (*fabp10a*) and Glutamate-Cysteine Ligase Catalytic Subunit (*gclc*), the cardiac toxicity gene ether-a-go-go related gene (*erg*), kidney toxicity marker connective tissue growth factor (*ctgf*), proliferative markers (*pcna* and *ccna2*) and tight junction protein gene (*claudin 5*) and brain plasticity marker (*cyp19a1b*) was normalized against the housekeeping elongation factor 1-alpha (*ef1a*) gene. The sequences of the primers are provided in Table 1.

### 2.11. Immunostaining

For immunohistochemistry experiments, vibratome sectioning were applied. Briefly, brains were rehydrated, washed in 1X PBS and included in agarose to be processed for vibratome sectioning (50 µm thickness). Sections were blocked in PBS-T containing 2% BSA. Next, sections were incubated with the following primary antibody overnight at 4 °C: mouse anti-PCNA (1:100; clone PC10, Dako; RRID: M0879). The sections were then washed and incubated with DAPI (4′,6′-diamidino-2-phenylindole) and secondary antibodies goat anti-mouse Alexa Fluor 594 or 488 for PCNA (1:300; REF: A11005 for Alexa 594 and A11001 for Alexa 488; Life Technologies; France; RRID: AB_ AB_2534073 and AB_ 2534069) for 2 h at room temperature. Sections were rinsed and the slides were mounted with Aqua-Poly/Mount (Polysciences). Note that PCNA antibody allows the detection of proliferative cells along the neurogenic niches, as previously described [59].

### 2.12. Investigation of BBB Permeability (Evans Blue Dye Injection)

Evans blue dye was used as a tracer to assess the permeability of the BBB [60]. Briefly, fish were anesthetized with 0.02% tricaine and intraperitoneally injected with 1% Evans blue dye diluted in PBS (10 µL of 1% Evans blue for 0.1 g of zebrafish body weight) before being placed back into the water. After 10 min, injected fish were sacrificed and heads were fixed with 4% PFA-PBS. Finally, the brains were dissected to be imaged.

### 2.13. Protein Extraction and Dot Blot

Zebrafish brains were lysed with TRIS HCl buffer (50 mM pH7.4 EDTA 0.01 mM) and centrifuged (10,000 rpm, 4 °C for 5 min). Supernatants were collected and frozen at −80 °C. Following the manufacturer’s protocol, protein concentration was determined according to the Bradford protein assay. For dot blot analysis, 20 µg of protein was placed on a nitrocellulose membrane. Following Ponceau Red staining, membranes were blocked with blocking buffer (5% milk in PBS containing 0.02% Tween 20) and incubated for 1 h 30 with rabbit anti-4-hydroxynonenal (4-HNE, a marker of oxidative stress) antibodies (Abcam; Paris, France; REF: ab46545). Membranes were then washed and incubated with secondary antibody coupled to HRP (1:2000) (Jackson’s Laboratories; Ely, United-Kingdom; goat anti-rabbit coupled with HRP, REF: JII035003) for 1 h 30 in order to be revealed with enhanced chemiluminescence substrates and imaged with the Amersham Imager 680 (Les Ulis, France).

### 2.14. Microscopy

Micrographs were obtained with an Eclipse 80i Nikon microscope (Champigny sur Marne, France) equipped with a Hamamatsu digital camera (Sciences, Tokyo, Japan), and with a Nanozoomer S60 (Hamamatsu, Massy, France). Pictures were adjusted for brightness and contrast in Adobe Photoshop. 

### 2.15. Cell Counting

The quantification of PCNA-positive specific regions from cryostat and vibratome sections (12 µm and 50 µm thickness/section for the cryostat and vibratome sections, respectively) allows the analysis of constitutive neurogenesis and proliferative activity. Images were analyzed for the detection of PCNA-positive nuclei using ImageJ software (National Institutes of Health, Bethesda, MD, USA; RRID: SCR_003070) after adjusting parameters (threshold, binary and watershed). Briefly, the parameters were set as follows for each picture: threshold 65, 255, particle size 200-infnity. Minor modifications in these parameters could be performed according to the experiments. In addition, ImageJ automated selection of PCNA-positive nuclei was manually double-checked and adjusted, if necessary, for each picture. Neuroanatomical structures were identified with DAPI counterstaining. Cell counting was performed in blind conditions by two different analysts, and the provided graphs correspond to the mean or percentage (%) of proliferative cells per section. The counting was performed on a total of 5 to 10 fish per condition, and the provided graphs correspond to the % mean of proliferative cells per section.

### 2.16. Instrumentation and LC-MS/MS Conditions

The total polyphenol content of the aqueous extract was determined by the Folin–Ciocalteu assay, as recently done by [31]. Polyphenols contained in the aqueous extract of *P. mauritianum* (0.25 g/L) were identified by ultra-high-performance liquid chromatography coupled with diode array detection and a HESI Orbitrap mass spectrometer (Q Exactive Plus, ThermoFisher; Dardilly, France). Briefly, 10 µL of sample was injected using an UHPLC system equipped with a Thermo Fisher Ultimate 3000 series WPS-3000 RS autosampler and then separated on a PFP column (2.6μm, 100 mm × 2.1 mm, Phenomenex, Torrance, CA, USA). The column was eluted with a gradient mixture of 0.1% formic acid in water (A) and 0.1% formic acid in acetonitrile (B) at the flow rate of 450 µL/min, with 5% B at 0.00 to 0.1 min, 75% B at 0.1 to 7.1 min, 95% B at 7.2 to 7.9 min and 5% B at 8.0 to 10 min. The column temperature was held at 30 °C and the detection wavelengths were set to 280 nm and 320 nm. 

For mass spectrometer parameters, heated electrospray ionization (HESI) was set to 2.8 kV, the capillary temperature was adjusted to 350 °C, the sheath gas flow rate was set to 60 units, the auxiliary gas flow rate was set to 20 units and the S-lens RF was equal to 50%. Mass spectra were registered in full scan mode from *m/z* 100 to 1500 in negative ion mode at a resolving power of 70,000 FWHM at *m/z* 400. The automatic gain control (AGC) was set at 1e^6^. Identification of the compounds of interest was based on their accurate mass, retention time, pattern fragmentation and commercial standards. Data were acquired using XCalibur 4.2.47 software (ThermoFisher; Dardilly, France) and processed by the Skyline 21.1 software (MacCoss Lab., Washington, DC, USA).

### 2.17. Statistical Analysis

Student’s *t*-test was performed for the comparisons between two groups. If more than two groups were analyzed, multiple testing was performed by one-way ANOVA. Error bars correspond to the standard error of the mean (SEM), and n values correspond to the number of animals or to the number of samples for all experiments. Note that for *p*-values, * *p* < 0.05; ** *p* < 0.01; *** *p* < 0.001 and **** *p* < 0.0001.

## 3. Results

### 3.1. Polyphenol Composition of the Aqueous Extract of P. mauritianum 

**Figure 1:**
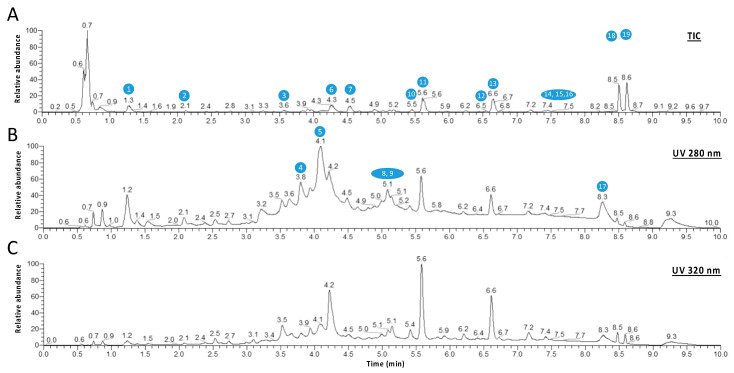
Spectra obtained for a representative *P. mauritianum* aqueous extract. (**A**) Representative total ion chromatogram (TIC) obtained in negative mode. (**B**) UHPLC-UV chromatograms obtained at 280 and 320 nm. (**C**) The different molecules are numbered according to their retention times. Note that numbers in blue correspond to the peak numbers reported in Table 2.

In a first step, the polyphenol composition of the aqueous extract of *P. mauritianum* was determined. First, the polyphenol content was assessed by the Folin–Ciocalteu method, providing 58.0 ± 7.2 mg GAE/g of plant dry powder. Then, the identification of compounds (mainly the polyphenols) from the aqueous extract of *P. mauritianum* (0.25 g/L) was performed using high-resolution mass spectrometry (HR-MS). *P. mauritianum* infusion contained a variety of polyphenols, including mainly gallic acid, caffeoylquinic acid, kaempferol derivatives, quercetin and its derivatives (Table 2, Figure 1). Of note, two triterpenes, asiatic and corosolic acids, were detected but not quantified (Table 2, Figure 1). 

### 3.2. Toxicity of the Aqueous Extract of P. mauritianum

**Figure 2:**
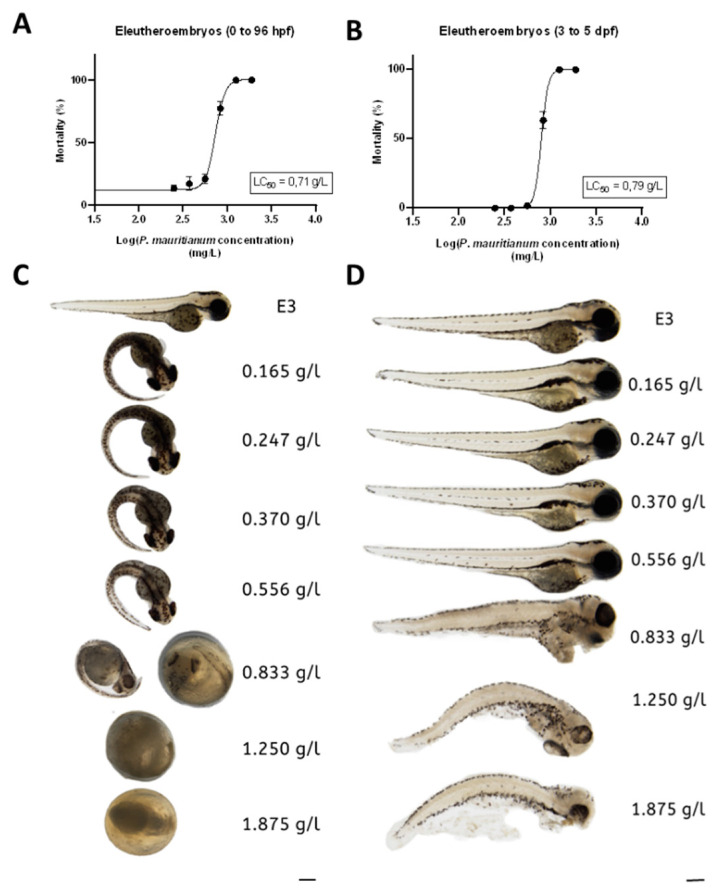
Determination of the lethal concentration _50_ (LC_50_) of the aqueous extract of *P. mauritianum* on eleutheroembryos. (**A**,**B**): Survival curve showing the mortality percentages of eleutheroembryos (3 hpf to 96 hpf and 3 dpf to 5 dpf, respectively) treated with 1.87 g/L, 1.25 g/L, 0.83 g/L, 0.55 g/L, 0.37 g/L, 0.24 g/L and 0.16 g/L of the aqueous extract from *P. mauritianum*, as a function of the log *P. mauritianum* concentrations. Note that most of the treated eleutheroembryos in A did not hatch. The LC_50_ values were 0.71 and 0.79 g/L in (**A**,**B**), respectively. (**C**,**D**): Representative pictures of eleutheroembryos at different concentrations at the end of the experimental assay (at 96 hpf for (**C**) and at 5 dpf for (**D**)). n = 60 eleutheroembryos for each tested concentration of three independent experiments (20 per experiment) for (**A**,**B**). Data are mean ± SD. Bar: 100 µm.

In order to study the toxicity of the aqueous extract of *P. mauritianum*, we first performed the well-recognized toxicity assay from the OECD guidelines 236 [58] on 3-h post-fertilization (hpf) to 96 hpf zebrafish eleutheroembryos. To this end, we incubated eleutheroembryos with a serial dilution of aqueous extract of *P. mauritianum* (1.87, 1.25, 0.83; 0.55, 0.37, 0.24 and 0.16 g/L). This treatment was renewed every day. Eleutheroembryos treated at 0.165 g/l to 0.556 g/l were alive and exhibited normal morphology according to OECD criteria, while, at 0.83 g/L and above, eleutheroembryos were coagulated (Figure 2A,C). Of interest, from 0.165 g/L to 0.556 g/L, most of the eleutheroembryos did not hatch. Given that this is not a parameter of toxicity according to the OECD criteria, the LC_50_ was estimated at 0.71 g/L.

In order to avoid any developmental toxicity, we decided to reproduce the same protocol on 3-days post-fertilization (dpf) to 5 dpf eleutheroembryos. From 3 dpf, zebrafish morphogenesis is almost complete and eleutheroembryos have a functional liver and kidney. Similar to the first test, at the highest concentrations (1.87 g/L, 1.25 g/L and 0.83 g/L), the aqueous extract led to the death of eleutheroembryos, while, at lower doses, they developed normally (Figure 2B,D). The LC_50_ was estimated to 0.79 g/L and appeared not significantly different from the one calculated in those treated from 3 hpf to 96 hpf (OECD assay). Consequently, the maximum non-toxic concentration (MNTC) that could be used in zebrafish eleutheroembryos was estimated at 0.556 g/L. We tested this concentration on adults and noticed immediately some behavioral abnormalities (low movement, hyperventilation) that were not observed at 0.25 g/L (data not shown). Thus, we decided to define 0.25 g/L as a safe dose for both eleutheroembryos and adult zebrafish, and to use this dose for further experiments.

**Figure 3:**
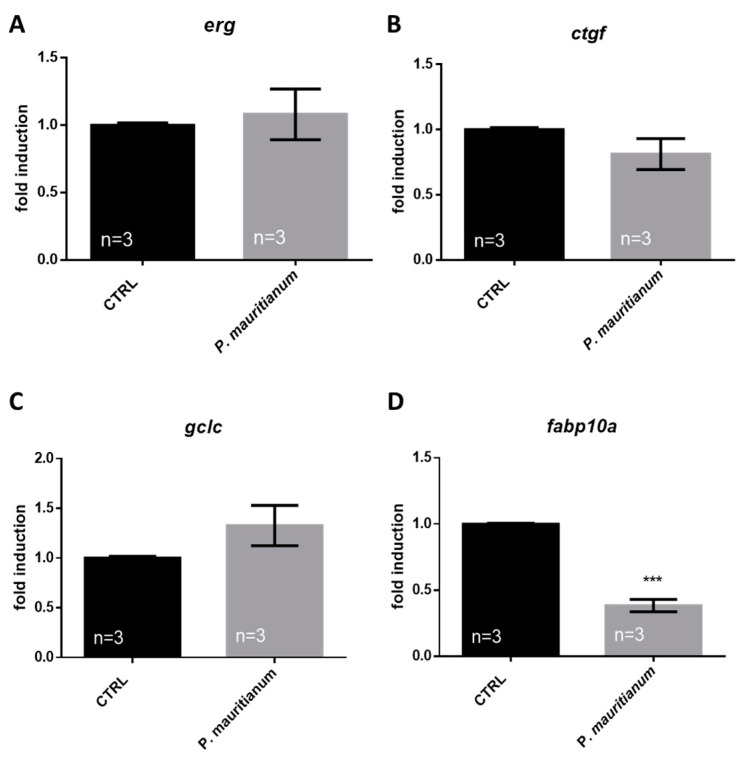
Effect of aqueous extract of *P. mauritianum* on specific markers of cardiac, renal and liver function. (**A**) RT-qPCR gene expression analysis of cardiac-specific gene *erg* (**A**), renal-specific gene *ctgf* (**B**) and liver-specific genes *gclc* and *fabp10a* (**C**,**D**), in CTRL and *P. mauritianum*-treated (3 to 5 dpf). n = 3 pools of 60 eleutheroembryos. Bar graph: standard error of the mean (SEM). Student’s *t*-test: *** *p* < 0.001.

In order to ascertain the safety of this dose, eleutheroembryos were treated from 3 to 5 dpf with the aqueous extract of *P. mauritianum* (0.25 g/L) and analyzed for the expression of specific genes involved in cardiac (*erg*), liver (*gclc* and *fabp10a*) and renal (*ctgf*) functions. The RT-qPCR results showed no significant changes in the expression of *erg, ctgf* and *gclc* (Figure 3A–C). However, *fabp10a* gene expression was significantly decreased in the treated larvae compared to controls (Figure 3D). This raises the question of the potential impact of the *fatty acid binding protein 10 a* (*fabp10a*) gene’s downregulation during development.

### 3.3. Aqueous Extract of P. mauritianum Exhibits Lipid-Lowering Effects in High-Fat-Diet-Fed Larvae 

**Figure 4:**
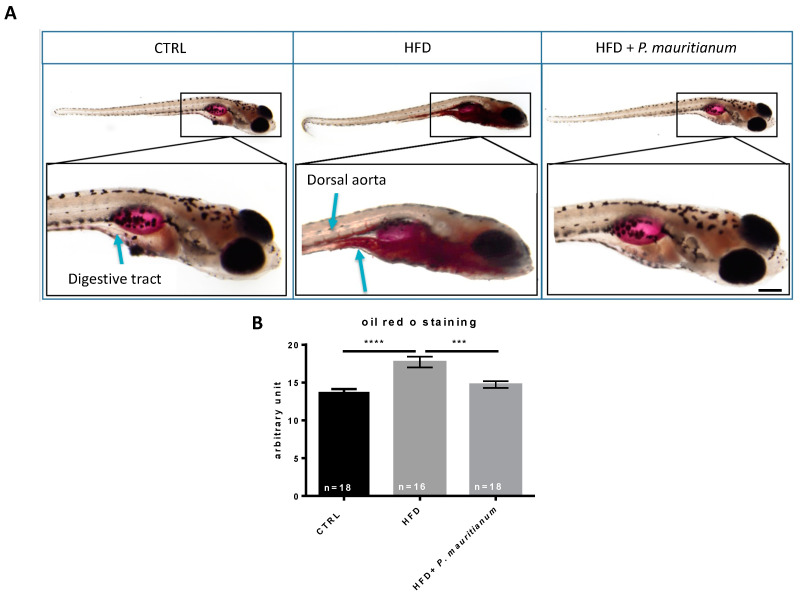
Aqueous extract of *P. mauritianum* prevents lipid accumulation in HFD larvae. (**A**) Representative pictures of ORO staining in CTRL, HFD and HFD + with *P. mauritianum* (0.25 g/L). (**B**) Bar graph showing ImageJ ORO staining quantification in CTRL, HFD and HFD + *P. mauritianum* demonstrating the efficiency of HFD treatment on lipid accumulation and the preventive effects of *P. mauritianum* on lipid accumulation. n = 16–18 larvae from 2 independent experiments. Bar graph: standard error of the mean (SEM). One-way ANOVA (**B**). *** *p* < 0.001; **** *p* < 0.0001. Bar: 100 µm for low-magnification and 400 µm for high-magnification larva image.

Considering the general absence of adverse effects of the aqueous extract of *P. mauritianum* at 0.25 g/L, we decided to further explore its potential therapeutical properties. This plant is traditionally suggested to exhibit anti-lipidemic effects and so to decrease cholesterol levels. We consequently aimed to investigate these metabolic properties using a model of high-fat-diet-fed larvae, as previously described [31]. To this end, we separated 7 dpf larvae into three groups: CTRL, HFD and HFD + *P. mauritianum* (0.25 g/L). The CTRL group was fed with normal dry food (Gemma 75) and incubated in E3 medium. The HFD group was fed with 0.1% egg yolk prepared in E3 medium, and the HFD + *P. mauritianum* group was fed with 0.1% egg yolk prepared in the aqueous extract of *P. mauritianum* (0.25 g/L). The medium and food were renewed every day until 11 dpf. At the end of the experimental procedure, Oil Red O staining (ORO) was performed to reveal neutral triglycerides and lipid accumulation (Figure 4). As expected, HFD larvae exhibited stronger and wider staining than controls in the digestive tract and also in the caudal vein, which were not stained in normal conditions (Figure 4A). In contrast, HFD larvae treated with *P. mauritianum* displayed weaker staining, similar to the CTRL group (Figure 4A). In order to further confirm this observational result, we performed quantification using ImageJ, showing significantly stronger ORO staining in HFD larvae, prevented by *P. mauritianum* treatment (Figure 4B). These experiments were repeated two times independently and led to similar results. 

Consequently, the aqueous extract of *P. mauritianum* prevented lipid accumulation in the HFD-fed larvae, suggesting anti-lipidemic properties. 

### 3.4. Preventive Effect of Aqueous Extract of P. mauritianum on Overfeeding-Induced Weight Gain in Adult Zebrafish 

**Figure 5:**
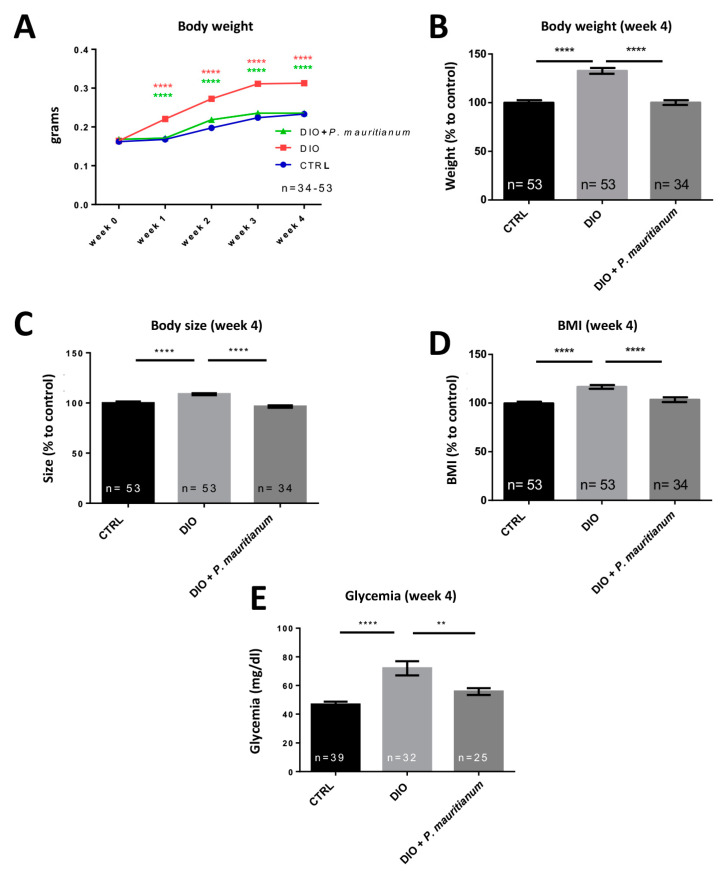
The aqueous extract of *P. mauritianum* significantly prevents body weight gain, increased BMI and hyperglycemia induced by DIO. (**A**) Line graph showing the increase in body weight during the experiment from week 0 to week 4 in CTRL, DIO and DIO + *P. mauritianum* (0.25 g/L) groups. (**B**–**E**) Graphs showing the body weight (**B**), size (**C**), BMI (**D**) and fasting blood glucose (**E**) of CTRL, DIO and DIO + *P. mauritianum* at the end of the experimental procedure (week 4). All the data result from three independent experiments providing the same results. n = 25–53, number of fish. Bar graph: standard error of the mean (SEM). Two-way ANOVA (**A**); one-way ANOVA (**B**–**E**): ** *p* < 0.01; **** *p* < 0.0001.

Considering the interesting properties of *P. mauritianum* in larvae, we decided to investigate its impact on a model of diet-induced obesity (DIO) previously established in adult zebrafish [29]. This model results in increased body weight, BMI and fasting blood glucose [29,54]. From the first week of overfeeding, DIO fish gained weight compared to the CTRL fish. Interestingly, the DIO + *P. mauritianum* prevented an increase in body weight (Figure 5A). Similar results were obtained until the fourth week (Figure 4B and Figure 5A). In addition, while the size of the DIO fish increased compared to CTRL ones, the DIO + *P. mauritianum* group’s size remained similar to fish fed a normal diet (Figure 5C). Similar results were obtained for the calculated BMI (Figure 5D). Interestingly, the aqueous extract of *P. mauritianum* also prevented a significant increase in fasting blood glucose resulting from 4 weeks of the DIO protocol (Figure 5E). Overall, these results showed that the aqueous extract of *P. mauritianum* exhibited a protective effect against weight gain, increased BMI and hyperglycemia induced by DIO.

**Figure 6:**
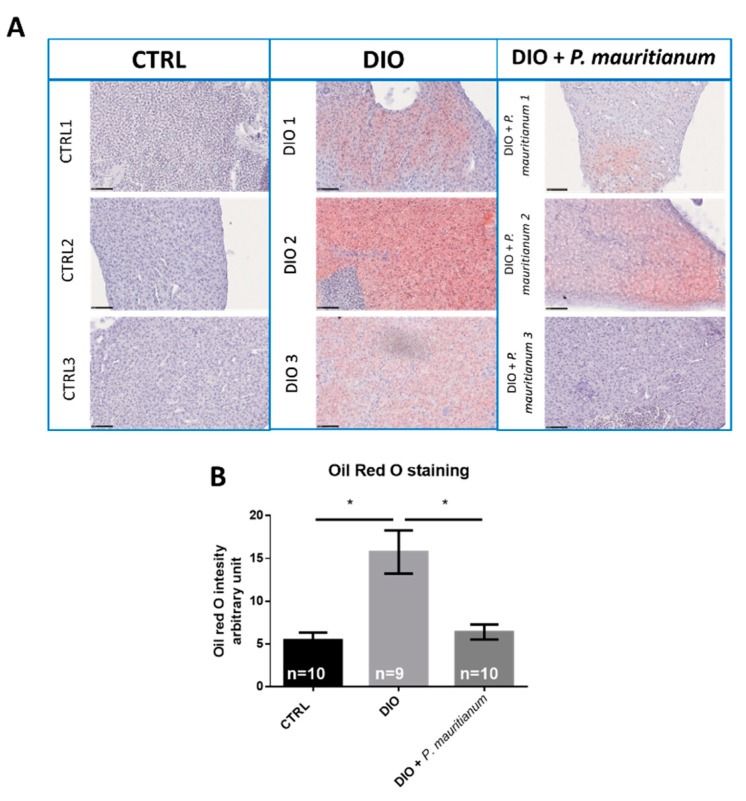
The aqueous extract of *P. mauritianum* prevents lipid accumulation in the liver of adult DIO fish. (**A**) Liver sections stained with ORO in three representative individual control fish (CTRL 1 to 3), three individual DIO fish (1 to 3) and three individual DIO fish treated with *P. mauritianum* (0.25 g/L; 1 to 3), showing no lipid accumulation, heterogeneous lipid (red) accumulation and low lipid accumulation (faint red), respectively, after the 4th week of the feeding protocol. (**B**) Red color intensity quantification using ImageJ quantification for mean red color intensity on the liver sections of CTRL, DIO and DIO treated with *P. mauritianum.* n = 9–10, number of fish. Bar graph: standard error of the mean (SEM). One-way ANOVA: * *p* < 0.05. Bar: 100 µm.

The liver is an important organ involved in lipid metabolism. Nonalcoholic fatty liver (NAFL) and liver steatosis are states of abnormal lipid metabolism that result in hepatic lipid accumulation and liver dysfunction. These conditions are associated with obesity and diabetes in humans as well as in many animal models, such as mice and zebrafish [8,43]. We consequently decided to study lipid accumulation by ORO staining in the liver of the CTRL, DIO and DIO + *P. mauritianum* groups. In DIO fish, we observed abnormal lipid accumulation in the liver (Figure 6A), while the livers of CTRL fish remained unstained. However, the ORO staining appeared heterogeneous among the DIO fish, showing differences in individual susceptibility to liver steatosis (Figure 6A). Interestingly, DIO fish treated with *P. mauritianum* showed only faint or undetectable ORO staining (Figure 6A). This result was reinforced by the evaluation of the ORO staining using a scoring system described in the Materials and Methods. As shown in Figure 6B, *P. mauritianum* exerted a preventive effect on lipid accumulation in the liver compared to the DIO fish.

### 3.5. P. mauritianum Prevents Brain Homeostasis Disruption Induced by Overfeeding

**Figure 7:**
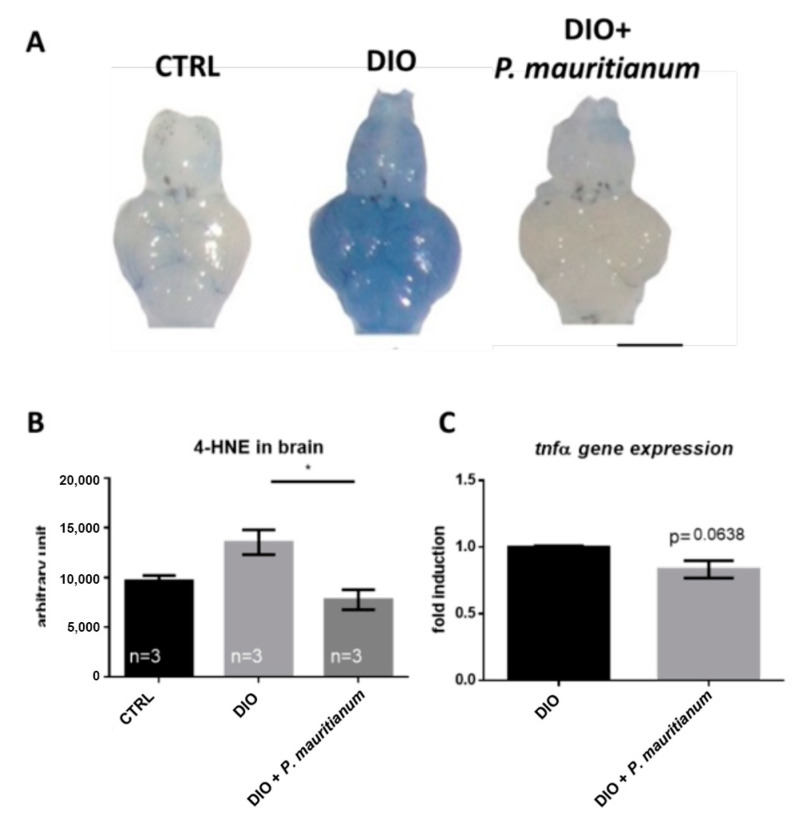
Aqueous extract of *P. mauritianum* prevents BBB leakage and brain oxidative stress. (**A**) Dorsal views of zebrafish brains after Evans blue dye injection in CTRL, DIO and DIO + *P. mauritianum*-treated fish. Zebrafish brains remained mostly white, except for the DIO brains. Note that in DIO, the blue staining is heterogeneous between fish. (**B**) Dot blot quantification showing that the *P. mauritianum* treatment in DIO fish prevented the cerebral increase in 4-HNE levels induced by overfeeding. (**C**) qPCR gene expression analysis of tnfα in DIO and DIO + P. mauritianum. n = 5–6 number of fish brains studied for (**A**), n = 3 pools of 2 brains for (**B**,**C**). Bar graph: standard error of the mean (SEM). Student’s *t*-test for (**B**,**C**): * *p* < 0.05. Bar: 0.8 mm.

We then investigated whether brain homeostasis was disrupted in overfeeding conditions. Indeed, DIO is known to impair blood-brain barrier (BBB) integrity and brain homeostasis even in fish [29,54]. We first investigated BBB function using the Evans blue dye, which is not able to cross the BBB under normal conditions. The brains of DIO fish appeared blue (with heterogeneity between animals) compared to the controls, whose brains remained white (Figure 7A). In addition, the treatment of fish with *P. mauritianum* limited the increase in brain 4-HNE levels, a marker of oxidative damage (Figure 7B). Previously, we demonstrated that DIO induced a weak increase in neuroinflammatory state [29]. Thus, we decided to compare some proinflammatory markers in the brains of DIO and DIO + *P. mauritianum* fish. The RT-qPCR analysis of the whole brain showed no striking effects on *tnfα* gene expression (Figure 7C). 

**Figure 8:**
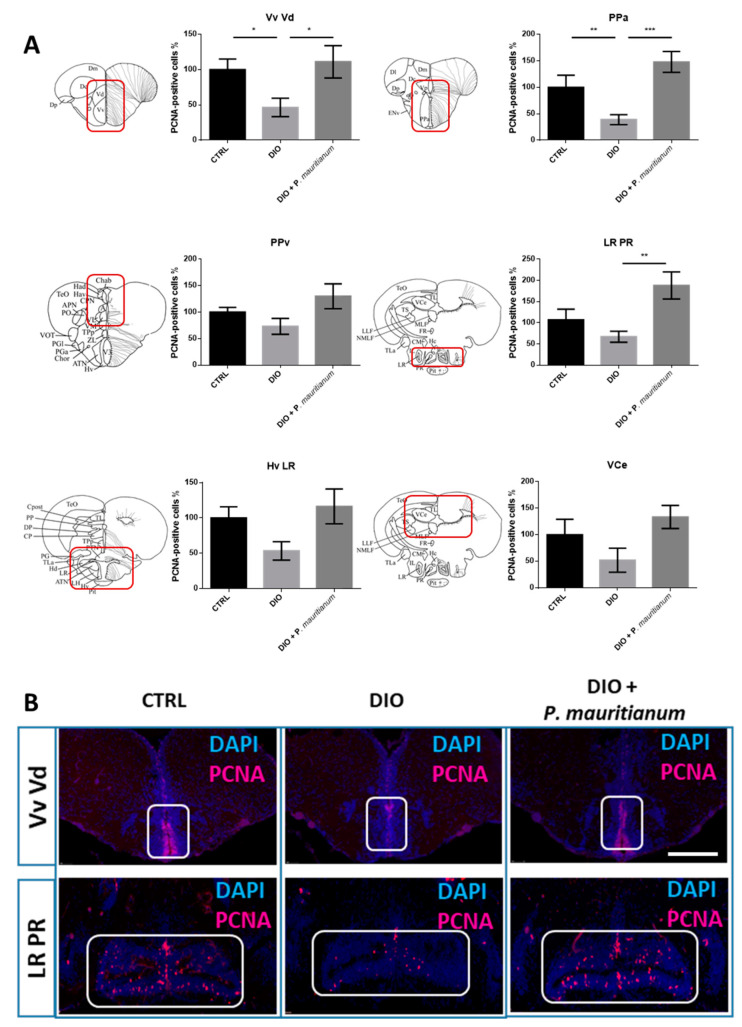
DIO reduced proliferation in the main neurogenic niches while *P. mauritianum* treatment rescued it. (**A**) Statistical analysis of the number of proliferative cells (PCNA-positive) in CTRL, DIO and DIO + *P. mauritianum*-treated zebrafish. The respective brain schemes correspond to the transversal sections of the zebrafish brain for each studied region, showing the main brain domains/nuclei according to the Zebrafish Brain Atlas from Wullimann et al., and were adapted from Pellegrini et al. [61,62]. DIO + *P. mauritianum* fish displayed neurogenesis similar to controls in the main neurogenic niches studied. (**B**) Representative pictures of PCNA immunohistochemistry (red) and cell nuclei counterstaining (DAPI in blue) on vibratome brain sections of CTRL (left) and DIO-treated fish (middle) and DIO + *P. mauritianum*-treated zebrafish (right). n = 5–10 number of brains studied pooled from two independent experiments. Bar graph: standard error of the mean (SEM). Student’s *t*-test: * *p* < 0.05; ** *p* < 0.01; *** *p* < 0.001. Bar = 150 μm. Vv: ventral nucleus of ventral telencephalic area; Vd: dorsal nucleus of ventral telencephalic area; PPa: parvocellular preoptic nucleus, anterior part; PPv: periventricular pretectal nucleus; Hv: ventral zone of periventricular hypothalamus; LR: lateral recess of diencephalic nucleus; PR: posterior recess of diencephalic ventricle; VCe: valvula cerebelli.

In addition, we decided to study the brain plasticity through the analysis of neurogenesis. The effects of DIO + *P. mauritianum* were analyzed in the main neurogenic niches by PCNA immunohistochemistry. In the brains of DIO fish, the number of PCNA-positive cells was generally decreased in the ventral and dorsal nuclei of the ventral telencephalon (Vv Vd), the anterior part of the preoptic area (PPa), the periventricular pretectal nucleus (PPv), the ventral zone of the periventricular hypothalamus and the lateral recess of the diencephalic nucleus (Hv LR), as well as along the lateral recess and posterior recesses of the diencephalic ventricle (LR PR) and the valvula cerebelli (VCe), compared to controls, as previously shown [29] (Figure 8). In contrast, DIO fish treated with the plant extract displayed a basal neurogenesis. Indeed, in the Vv Vd, the PPa and the LR PR regions, *P. mauritianum* increased significantly the neurogenesis compared to that observed in DIO fish, and the same trend was observed in the remaining regions of the brain (PPv, HvLR and VCe).

Taken together, the overfed fish treated with the plant extract did not show striking brain alterations at the level of the BBB, oxidative stress and neurogenesis, in contrast to untreated overfed fish. 

### 3.6. Effect of P. mauritianum on Feeding Behavior and Feces Production

**Figure 9:**
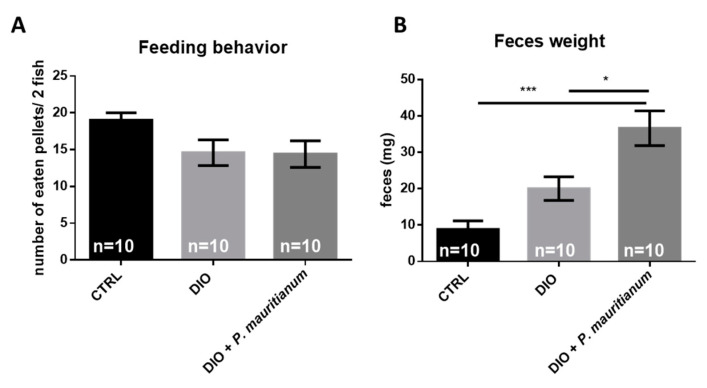
*P. mauritianum* does not impact feeding behavior and favors feces excretion. (**A**) Graph showing the number of pellets consumed by fish for 5 days in CTRL, DIO and DIO treated with *P. mauritianum* groups. (**B**) Quantification of the feces weight excreted by the fish for 5 days in the CTRL, DIO and DIO treated with *P. mauritianum* groups. n = 10, number of zebrafish used in the experiment for 5 days (2 fish/tank/day). The feeding behavior in (**A**) was assessed for fish each day, and in (**B**) the feces excretion was measured for 10 fish. Bar graph: standard error of the mean (SEM). One-way ANOVA: * *p* < 0.05; *** *p* < 0.001.

These protective effects of *P. mauritianum* suggest that DIO fish treated with *P. mauritianum* could display altered feeding behavior. This would consequently result in no weight gain, no increase in BMI, normal fasting blood glucose and normal liver physiology. To test this hypothesis, we performed a feeding quantification by applying visible and countable feeding pellets to each group, and then compared the number of pellets eaten by each group. The DIO fish and the DIO + *P. mauritianum* fish did not display any significant change in feeding behavior (Figure 9A), supporting our visual observations of active feeding when we fed both groups of fish nine times a day. This indicates that the fish ate the same amount of food and that the preventive effects of *P. mauritianum* were not due to reduced calorie intake. However, we noticed that the quantity of feces excreted by DIO fish treated with *P. mauritianum* was increased compared to DIO fish. After weighing the feces produced by each group during the night for 5 days, we obtained significantly higher feces production in the DIO fish treated with *P. mauritianum* than in the DIO and CTRL groups (note that a Student *t*-test comparing the feces production between the CTRL and DIO groups showed a significant increase in feces production in DIO) (Figure 9B). 

### 3.7. Aqueous Extract of P. mauritianum Has No Impact on Brain Homeostasis under Normal Chow Feeding Conditions

**Figure 10:**
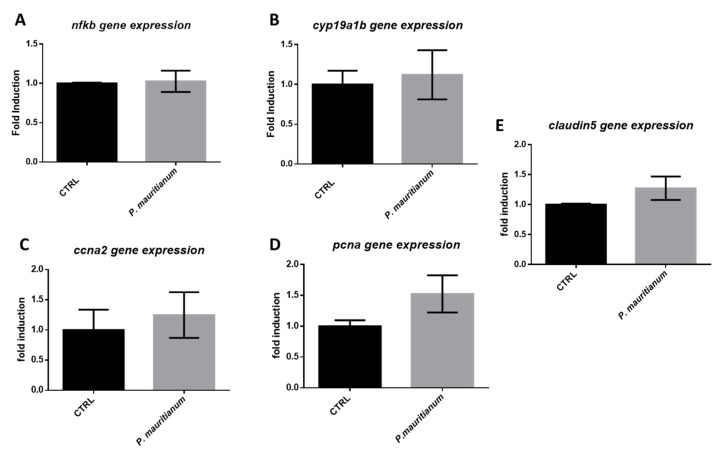
*P. mauritianum* has no effect on brain homeostasis in normal feeding conditions**.** RT-qPCR gene expression analysis of nuclear factor kappa B (*nfκb*) a transcription regulator of inflammation (**A**), aromatase B gene (*cyp19a1b*) a brain plasticity marker (**B**), cyclin-a2 gene (*ccna2*) and proliferating cell nuclear antigen (*pcna*) brain, two cell proliferation markers (**C**,**D**), and claudin 5, a tight junction gene involved in blood-brain barrier integrity (*claudin 5*) (**E**). n = 3 pools of 2 brains. Bar graph: standard error of the mean (SEM). Student’s *t*-test.

Aiming to test the effect of *P. mauritianum* on the brain, regardless of its effect on preventing the increase in the body weight, we decided to normally feed zebrafish and treat them overnight for 3 days with *P. mauritianum*, in order to analyze by RT-qPCR the expression of genes involved in BBB function, inflammation, brain plasticity and brain cell proliferation. The first group (CTRL) was treated with water, while the second was treated with an aqueous extract of *P. mauritianum* (0.25 g/L) overnight for 3 days. At the end of this experimental procedure, no change in body weight was observed (Figure S1) and fish behaved normally. The brains were extracted and processed for gene expression analysis. We did not observe any significant change in the gene expression of the inflammatory transcription factor (*nfκb),* tight junction protein (*claudin 5)*, brain plasticity marker (*cyp19a1b*) and proliferative marker (*pcna* and *ccna2*) (Figure 10). These data reinforce the hypothesis that the preventive effect of *P. mauritianum* on the brains of overfed fish was due to the prevention of body weight increase and hyperglycemia and not to the effects of *P. mauritianum* polyphenols and their metabolites on the brain.

### 3.8. Therapeutic Effects of Aqueous Extract of P. mauritianum on Weight Gain 

**Figure 11:**
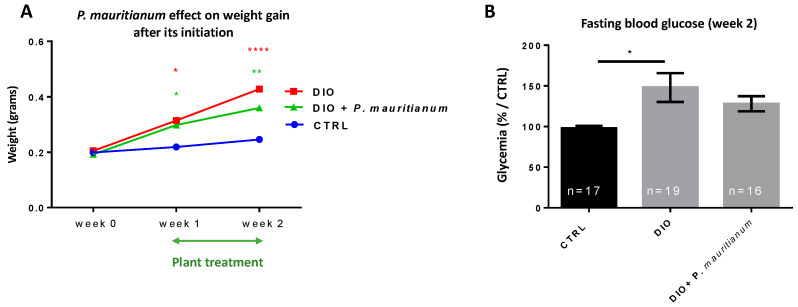
Aqueous extract of *P. mauritianum* limited the increase in body weight and glycemia after the onset of weight gain. (**A**) Line graph showing the increase in the body weight during the experiment from week 0 to week 2 in the CTRL, DIO and DIO + *P. mauritianum* groups. The green “double arrow” represents the plant treatment from week 1 to week 2. (**B**) Bar graph showing fasting blood glucose of CTRL, DIO and DIO + *P. mauritianum* at the end of the experimental procedure (week 2). n = 16–19, number of fish. Bar graph: standard error of the mean (SEM). Two-way ANOVA (**A**); one-way ANOVA (**B**): * *p* < 0.05; ** *p* < 0.01; **** *p* < 0.0001.

We next considered whether the aqueous extract of *P. mauritianum* could display a therapeutic effect. For this purpose, all fish were fed according to the DIO protocol for one week without any plant treatment. As shown in Figure 11A, the body weight of DIO fish increased significantly compared to control fish. After this one-week period of overfeeding, a subgroup of DIO fish was treated with *P. mauritianum* for one additional week, still in the overfeeding condition (DIO + *P. mauritianum*), while the other subgroup was simply overfed (DIO). The body weights of the DIO and DIO + *P. mauritianum*-fed fish were significantly higher than those of the control group. However, the weight gain of *P. mauritianum*-treated fish was less pronounced than for DIO. The fasting blood glucose measured at week 2 showed 50% higher glycemia in the DIO compared to CTRL fish. The DIO + *P. mauritianum*-treated fish exhibited a 30% increase in glycemia compared to controls (but this was not significant). Moreover, the fasting blood glucose levels in the DIO + *P. mauritianum* group tended to be lower than in DIO-treated fish only (remaining non-significant) (Figure 11B). Thus, *P. mauritianum* limited the sharp increase in glycemia. In the brain, no significant change in cell proliferation was observed among all groups (Figure S2). 

## 4. Discussion

*P. mauritianum* is a plant widely used in the Mascarene Islands (Mauritius and Reunion Islands) for its traditional benefits, considering its anti-cholesterol and “anti-diabetic” effects [32,33]. Recent scientific experiments on this medicinal plant have demonstrated its anti-bacterial, antiviral, anti-inflammatory and antioxidant properties [25,26,63,64]. In this study, we investigated for the first time the in vivo toxicity of an aqueous extract of *P. mauritianum* according to the OECD guidelines, using a zebrafish model. We determined a maximum non-toxic concentration of 0.25 g/L on larvae and adults and showed that this dose has interesting lipid-lowering properties in HFD larvae and an anti-weight-gain effect in overfed adult fish. This dose also prevents the deleterious impact of overfeeding on peripheral metabolic parameters (BMI, glycemia, liver steatosis) and also on the brain, probably through the absence of development of metabolic disruption in overfed fish treated with *P. mauritianum* extract. Very interestingly, our data also demonstrate that treated fish have increased feces production. We speculate that this is due to changes in gut microbiota and intestinal absorption, which should be confirmed with more investigations. 

### 4.1. Composition of Aqueous Extract of Polyphenols

Performing LC-MS/MS, we observed that the aqueous extract of *P. mauritianum* contained many polyphenols, including mainly gallic acid, caffeoylquinic acid, kaempferol derivatives, quercetin and its derivatives. Moreover, two interesting triterpenoids, asiatic and corosolic acids, were also present in the *P. mauritianum* extract. Our laboratory previously demonstrated that other medicinal plants contained caffeic acid, caffeoylquinic acid (i.e., chlorogenic acid), dicaffeoylquinic acid and some kaempferols, namely hexoside and quercetin hexoside, as well as their respective derivatives, for *A. borbonica* [27,65], and 5-caffeoylquinic acid (chlorogenic acid), quercetin glycosides and kaempferol glycosides and their respective derivatives for *H. lanceolatum* [31], and most of these compounds have also been detected in other medicinal plants [25]. We can speculate that *P. mauritianum*’s richness in gallic acid and in the two particular triterpenoids (asiatic and corosolic acids) could be responsible for the properties of this plant regarding metabolic disorders [66,67]. However, we require further investigations to confirm this hypothesis.

### 4.2. Toxicity of the Aqueous Extract of P. mauritianum

Our toxicity assay following the guidelines of the fish acute toxicity assay from the OECD gave us similar LC_50_ values of 0.71 and 0.79 g/L for fish treated from 3 hpf to 96 hpf and 3 dpf to 5 dpf, respectively. The maximum non-lethal concentration determined in toxicity assays was theoretically around 0.5 g/L. However, at this concentration, eleutheroembryos (3 hpf to 96 hpf) did not hatch and behavioral changes have been observed in adults. Considering this point, we performed our experiments at 0.25 g/L during the developmental and adult stages. Indeed, this dose exhibited no apparent signs of disturbance in both eleutheroembryos and adults (i.e., normal behavior and feeding). In addition, the gene expression of most genes involved in cardiac, hepatic and renal functions remained at normal levels in eleutheroembryos. Nevertheless, we observed a significant decrease in *fabp10a* gene expression (fatty acid binding protein 10a or liver bile acid binding protein), which is responsible for the intracellular binding and trafficking of long-chain fatty acids to the liver and is sometimes used as a hepatic toxicity marker [68,69,70]. However, some data also document its expression in the ventral endoderm during development [68], raising the question of the cells downregulating its expression following *P. mauritianum* treatment during embryogenesis. It could be interesting to further investigate the protein expression and function of this fatty acid binding protein in the clearance of lipids and their metabolism under *P. mauritianum* treatment to ascertain its non-toxic effects in developmental and adult stages. Of interest, this fatty acid binding protein is also expressed in the intestine of adult zebrafish and could potentially be involved in lipid absorption [71].

### 4.3. Effects of the Aqueous Extract of P. mauritianum on Metabolic Parameters

The dose of 0.25 g/L limited lipid accumulation in HFD larvae (i.e., digestive tract + blood vessels and liver) and in overfed adult fish (i.e., liver). These findings support the traditional lipid-lowering effect of *P. mauritianum*. Indeed, this medicinal plant is supposed to increase cholesterol elimination from the body, and recent work also showed a decrease in oxidized LDL uptake by macrophages under *P. mauritianum* treatment [21,26,32]. Of interest, *P. mauritianum* exerts striking effects on overfed adult zebrafish (DIO protocol) by preventing weight gain, increased BMI, hyperglycemia and liver steatosis, and consequently improving almost all deleterious effects induced by overfeeding. Indeed, the body weight of DIO fish treated with the plant extract followed the same trend as the CTRL group during the 4 weeks of the experiment. This result is not the consequence of a modification in feeding behavior, as DIO + *P. mauritianum* fish ate the same amount of food as DIO fish. Moreover, in a therapeutic approach, we demonstrated that after 1 week of overfeeding and the establishment of weight gain, a treatment with *P. mauritianum* partly reduced weight gain and normalized glycemia. Such an effect was also observed with another medicinal plant, *H. lanceolatum*, rich in quercetin, kaempferol and chlorogenic acid [31]. Thus, this result raises the question of the compounds behind this effect and suggests the potential role of common polyphenols found in *H. lanceolatum* and *P. mauritianum*, such as quercetin, kaempferol and their respective derivatives.

### 4.4. Aqueous Extract of P. mauritianum: Polyphenol Content, Lipid Absorption/Metabolism and Microbiota

DIO fish treated with *P. mauritianum* display higher production of feces compared to DIO fish. This suggests that *P. mauritianum* could affect lipid absorption and the gut microbiota in overfeeding conditions. This is particularly interesting given that the aqueous extract of *P. mauritianum* is enriched in polyphenols, and that polyphenols modulate microbiota and nutrient absorption/metabolism [72]. According to the LC-MS/MS analysis for polyphenol identification, the aqueous extract of *P. mauritianum* is rich in gallic acid, caffeoylquinic acid, kaempferol hexoside and derivatives, as well as in quercetin and derivatives, corroborating data obtained in previous work [25]. 

Interestingly, the kaempferols tested on high-fat-diet-fed obese mice were capable of reverting the disorders resulting from obesity, including body weight, glucose intolerance and fat accumulation [73], similarly to what we observed by preventive treatment with *P. mauritianum* in DIO zebrafish. More interestingly, fecal microbiota analysis showed the capability of kaempferol treatment to alleviate the gut dysbiosis resulting from obesity [73]. This anti-obesity effect displayed by kaempferols on the high-fat-diet mouse model, and its mechanism of action, resembles strongly the anti-obesity effect of *P. mauritianum* extract treatment in the zebrafish model of DIO. In addition, several studies confirm the role of quercetin in ameliorating the dysbiosis state resulting from abdominal obesity [74], and in reducing insulin resistance in diet-induced models [75]. In vitro and in vivo studies indicate that gallic acid could positively affect the gut microbiota and decrease the growth of pathogenic bacteria [76]. It was also shown that gallic acid improves glucose tolerance, lipid metabolism, adipocyte hypertrophy and inflammation in obese mice [77,78]. Finally, Rangasamy and collaborators identified two triterpenoid molecules present in *P. mauritianum*, corosolic and asiatic acids [34], that were also identified in our LC-MS/MS analysis (Table 2). Interestingly, asiatic acid was shown to limit the deleterious effects of obesity and diabetes in diabetic mice and HFD rats [66,67].

Together, the anti-obesity effect of *P. mauritianum* extract could be exerted by a synergy of its main polyphenols acting on the gut microbiota, lipid absorption and metabolism. It will be interesting to further explore the impact of *P. mauritianum* extract on (i) the reduction of lipid intestinal transport, (ii) the reduction of plasma cholesterol, lipoproteins and triglycerides and (iii) their elimination. Moreover, the protective effect of *P. mauritianum* against the increased glycemia in overfed fish could reflect the “anti-diabetic” property of this traditional plant. This property is probably achieved through the prevention of weight gain and liver steatosis, which both serve to enhance the prediabetic state and increase glycemia [79,80]. Further investigations are now necessary to determine the polyphenol or polyphenol cocktail behind the beneficial properties of *P. mauritianum* on weight gain in our model (gallic acid, kaempferols, corosolic and asiatic acids).

### 4.5. Aqueous Extract of P. mauritianum and Brain Homeostasis

Under overweight and obesity conditions, brain homeostasis is impaired in both mammals and zebrafish [29,54,81]. We and others have previously demonstrated that DIO disrupts brain homeostasis by inducing BBB breakdown, cerebral oxidative stress and neuro-inflammation associated with impaired neurogenesis [29,39,40,53,54,82,83]. In our study, we confirmed this decreased brain plasticity observed in overfeeding conditions. Interestingly, overfed fish treated with *P. mauritianum* have a “normal” neurogenesis, BBB function and cerebral redox balance. The antioxidant capacity and protective effect of *P. mauritianum* extract against oxidative stress have been documented in vitro and are supported by bioactive polyphenols as phenolic acids and flavonoids [25]. In rat models of metabolic disorder (hypercaloric diet), gallic acid improved neurological parameters (memory, redox status) that were disrupted by the diet [84]. However, the potential beneficial effects of *P. mauritianum* on CNS protection is probably due to the absence of metabolic disorders in this overfed fish model. Indeed, the treatment of normally fed zebrafish with the plant extract did not impact the expression of genes involved in BBB function, inflammation and brain cell proliferation. Consequently, the preventive effect of the plant’s extract on the brain could be due to its indirect effect on the prevention of obesity development and its subsequent consequences (i.e., hyperglycemia), which are factors that disrupt brain homeostasis [29,55].

### 4.6. Suggested Mechanisms of Action for Aqueous Extract of P. mauritianum

In this work, the well-known traditional lipid-lowering properties of *P. mauritianum* were confirmed in our different models. It is now important to understand the mechanisms behind such an interesting effect. Among the possible explanations for the mechanistic pathways, the action of polyphenols on the increase in lipid metabolism should be studied. Flavonoids (i.e., quercetin and kaempferols, present in significant amounts in our plant extract) are known to regulate genes involved in lipid metabolism, such as SREBP-1, C/EBPα, PPARγ and PPARα [85,86]. Specifically, in zebrafish, studies have shown that kaempferols have anti-adipogenic properties, regulating several adipogenic factors such as C/EBPβ [86]. PPARα, highly expressed in the liver, has been shown to be involved in lipid catabolism and the β-oxidation of fatty acids [87]. Our data clearly show a decrease in lipid accumulation in the livers of adult overfed zebrafish treated with *P. mauritianum*. In parallel, preliminary data on zebrafish larvae indicate lower expression levels of the *fabp10a* gene, which is a fatty acid binding protein. This could lead to the hypothesis that the flavonoid-rich plant extract increases PPARα levels in the liver, thereby increasing fatty acid oxidation and decreasing lipid storage in the liver. In addition, the main compounds of *P. mauritianum* (gallic acid and kaempferols) have been presented in the literature as having an anti-obesity effect by attenuating the intestinal dysbiosis resulting from obesity [73,76]. Gallic acid treatment in HFD mice was shown to improve metabolic parameters such as glucose intolerance and to increase thermogenesis in the adipose tissue [88]. Together, these different compounds could reduce systemic inflammation and oxidative stress and prevent white adipose tissue expansion.

## 5. Conclusions

We determined the non-toxic dose of the aqueous extract of *P. mauritianum* (0.25 g/L) in eleutheroembryos and adult zebrafish. In modern drugstores and traditional medicine, 1 to 5 g of dried leaves should be infused in 1 L of water for 10–15 min, and consumed during the day. Considering that only a small percentage of total polyphenolic compounds may be absorbed in the small intestine, and considering the metabolism of polyphenols, we can assume that the dose required in zebrafish is higher than in humans. In zebrafish, we demonstrated, for the first time to our knowledge, that the 0.25 g/L dose shows significant lipid-lowering effects in HFD-treated larvae and overfed adults. Similarly, *P. mauritianum* at 0.25 g/L displays a preventive effect against the increase in body weight, glycemia and liver steatosis following overfeeding. It also improves metabolic parameters such as weight and fasting blood glucose after the onset of body weight gain. Therefore, our data confirmed that *P. mauritianum* extract could be an interesting “lipid-lowering” and “anti-diabetic” treatment. In addition, *P. mauritianum* has also an impact on feces production. Future work should further explore its role on gut functions (lipid absorption and metabolism) and on the gut microbiota. It would be consequently crucial to further investigate such properties on preclinical models of obesity and/or diabetes, such as HFD or *db*/*db* mice, in order to better understand the mechanisms and compounds responsible for these interesting properties of *P. mauritianum*.

## Figures and Tables

**Table 1:** Zebrafish qPCR primer sequences.

Gene	Forward Primer	Reverse Primer	Gene Accesion Number
*ef1α*	AGCAGCAGCTGAGGAGTGAT	CCGCATTTGTAGATCAGATGG	ENSDARG00000039502
*erg*	CAGATGCTCCGTGTGAAAGA	TGCGGTTCAGATGAAGACAG	ENSDARG00000077304
*fabp10a*	CCAGTGACAGAAATCCAGCA	GTTCTGCAGACCAGCTTTCC	ENSDARG00000038439
*gclc*	AAAATGTCCGGAACTGATCG	AACGTTTCCATTTTCGTTGC	ENSDARG00000013095
*tnfα*	GCGCTTTTCTGAATCCTACG	TGCCCAGTCTGTCTCCTTCT	ENSDARG00000009511
*claudin 5*	TCCTGGGTCTGATCCTGTG	CTCGATGAAGGCGGTGAC	ENSDARG00000043716
*ccna2*	AAAGCAGCTAACAACAGGACAGT	GGTTTACACGCAATTATCTGTGG	ENSDARG00000011094
*cyp19a1b*	TCGGCACGGCGTGCAACTAC	CATACCTATGCATTGCAGACC	ENSDARG00000098360
*pcna*	GGACAGAGGAGTGGCTTTGG	CTCACAGACCAGCAACGTCG	ENSDARG00000054155
*ctgf*	CTCCCCAAGTAACCGTCGTA	TCCACCAAACACACAAGTGG	ENSDARG00000042934

**Table 2:** Identification and quantification of compounds from aqueous extract of *P. mauritianum* (0.25 g/L) by LC-MS/MS analysis. Spectra analysis was also performed (upper spectrum: representative total ion chromatogram (TIC) obtained in negative mode; lower spectrum: HPLC-UV chromatogram obtained at 280 and 320 nm). Results are expressed as mean in ng/mL. RT, retention time. Nq, not quantified. Note that numbers correspond to the ones shown on the different spectra from Figure 1 (peak numbers reported in the table). Note that some isomers were detected (e.g., kaempferol hexoside).

Peak Number	RT (min)	Compound	[M-H]-	MS/MS Fragments	Concentration *P. mauritianum* Infusion (ng/mL)
**1**	1.3	Gallic acid	169.0137	125.0236	172.3 ± 7.5
**2**	2.1	Protocatechuic acid	153.019	109.0287	5.6 ± 0.4
**3**	3.6	Caffeoylquinic acid	353.0886	191.0558	32.3 ± 2.1
**4**	3.8	Caffeic acid	179.0346	135.0445	1.6 ± 0.1
**5**	4.1	Caffeoylquinic acid	353.0886	191.0558	8.9 ± 1.1
**6**	4.3	Coumaroylquinic acid	337.0936	191.0558, 173.0452, 93.0336	*nq*
**7**	4.5	Quercetin hexoside	463.0882	300.0282	0.3 ± 0.0
**8**	5.1	Quercetin hexoside	463.0882	300.0282	18.2± 0.6
**9**	5.1	Quercetin-O-(acetyl-hexoside)	505.099	300.0282	2.5 ± 0.0
**10**	5.5	Kaempferol hexoside	447.0940	284.0332	3.7 ± 0.1
**11**	5.6	Kaempferol hexoside	447.0940	284.0332	32.9 ± 0.1
**12**	6.5	Quercetin-O-(acetyl-hexoside)	505.099	300.0282	8.3 ± 0.5
**13**	6.6	kaempferol-O-(acetyl-hexoside)	489.1040	284.0333, 429.0836, 447.095	20.9 ± 0.0
**14**	7.4	Kaempferol-O-(acetyl-rhamnoside)	473.1090	284.0333, 413.0884	3.4 ± 0.0
**15**	7.5	Quercetin	301.0360	151.0029, 178.9981, 121.0285	2.0 ± 0.5
**16**	7.6	Kaempferol-O-(acetyl-rhamnoside)	473.1090	284.0333, 413.0884	2.0 ± 0.0
**17**	8.3	Kaempferol	285.0411	151.0032	2.1 ± 0.0
**18**	8.4	Asiatic acid	487,344	_	*nq*
**19**	8.6	Corosolic acid	471,3488	_	*nq*

## Data Availability

The data presented in this study are available on request from the corresponding author. The data is not publicly available due to administrative reasons.

## Supplementary Materials

The following supporting information can be downloaded at: https://www.mdpi.com/article/10.3390/antiox11071309/s1, Figure S1: Body weight of CTRL and *P. mauritianum* treated fish; Figure S2: Brain cell proliferation in the main neurogenic niches.
