# Supplementary material for: Aqueous Extract of Psiloxylon mauritianum, Rich in Gallic Acid, Prevents Obesity and Associated Deleterious Effects in Zebrafish
Source: Antioxidants (Basel). 2022 Jun 30;11(7):1309. doi: 10.3390/antiox11071309 (PMC9312056; doi:10.3390/antiox11071309)
Supplement: Supplementary file 1 [file antioxidants-11-01309-s001.zip › antioxidants-1769074-supplementary.pdf]

antioxidants

Supplementary Material: Aqueous Extract of *Psiloxylon mauritianum*, Rich in Gallic Acid, Prevents Obesity and Associated Deleterious Effects in Zebrafish

Batoul Ghaddar [1], Laura Gence [1,†], Bryan Veeren [1,†], Matthieu Bringart [1], Jean-Loup Bascands [1], Olivier Meilhac [1,2] and Nicolas Diotel [1,*]

[1] Université de La Réunion, INSERM, UMR 1188, Diabète Athérothrombose Thérapies Réunion Océan Indien (DéTROI), Saint-Denis, 97400 La Réunion, France; batoul.ghaddar@univ-reunion.fr (B.G.); laura.gence@univ-reunion.fr (L.G.); bryan.veeren@univ-reunion.fr (B.V.); matthieu.bringart@inserm.fr (M.B.); jean-loup.bascands@inserm.fr (J.-L.B.); olivier.meilhac@univ-reunion.fr (O.M.)
[2] CHU de La Réunion, Saint-Denis, 97400, La Réunion, France
* Correspondence: nicolas.diotel@univ-reunion.fr
† These authors contribute equally to this work.

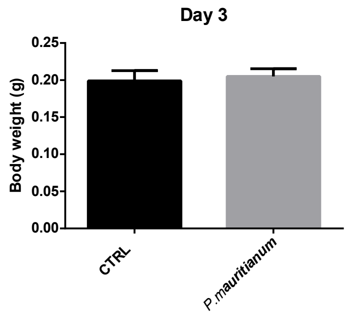

Supplementary Figure S1: Body weight of CTRL and *P. mauritianum* treated fish

After three days of treatment with water (CTRL) or *P. mauritianum* (0.25 g/L), no significant change in the body weight of CTRL and *P. mauritianum* treated fish was observed.

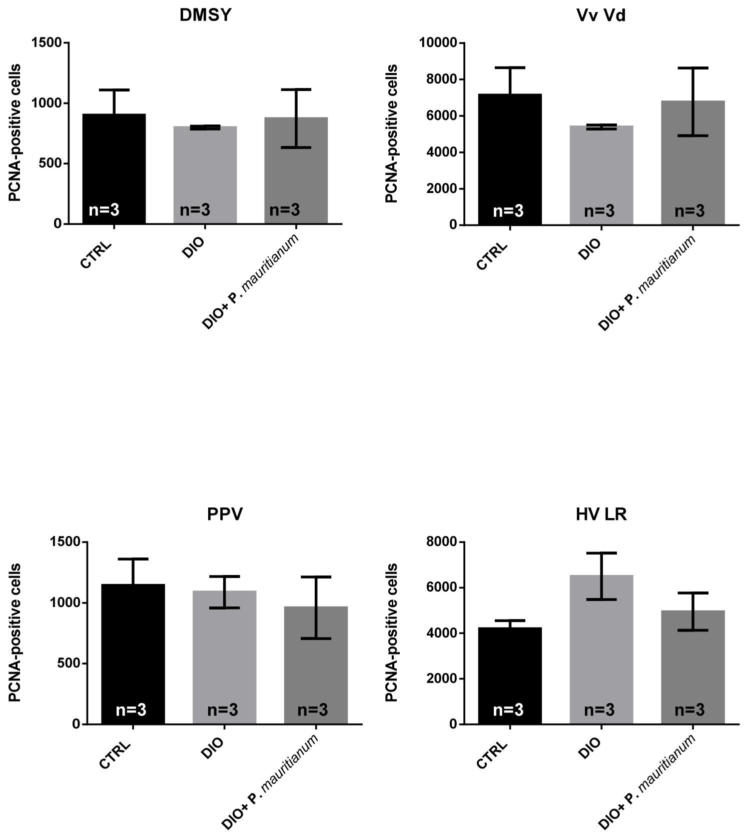

Supplementary Figure S2: Bran cell proliferation in the main neurogenic niches

In the therapeutic protocol, neurogenesis was not different between control, DIO and DIO + *P. mauritianum* at week 2 of the experiment for CTRL, DIO, DIO+ *P. mauritianum* treated fish in the main neurogenic niches studied.
